# Dynamic changes of small RNAs in rice spikelet development reveal specialized reproductive phasiRNA pathways

**DOI:** 10.1093/jxb/erw361

**Published:** 2016-10-04

**Authors:** Qili Fei, Li Yang, Wanqi Liang, Dabing Zhang, Blake C. Meyers

**Affiliations:** ^1^Department of Plant & Soil Sciences and Delaware Biotechnology Institute, University of Delaware, Newark, DE 19711, USA; ^2^State Key Laboratory of Hybrid Rice, Joint International Research Laboratory of Metabolic and Developmental Sciences, Shanghai Jiao Tong University and University of Adelaide Joint Centre for Agriculture and Health, School of Life Sciences and Biotechnology, Shanghai Jiao Tong University, Shanghai 200240, China; ^3^School of Agriculture, Food and Wine, University of Adelaide, South Australia 5064, Australia; ^4^Donald Danforth Plant Science Center, 975 North Warson Road, St. Louis, MO 63132, USA; ^5^University of Missouri – Columbia, Division of Plant Sciences, 52 Agriculture Lab, Columbia, MO 65211, USA

**Keywords:** Anther, Argonaute, microRNA, phasiRNA, rice, spikelet.

## Abstract

Dissection of the genetic pathways and mechanisms by which anther development occurs in grasses is crucial for both a basic understanding of plant development and for examining traits of agronomic importance such as male sterility. In rice, MULTIPLE SPOROCYTES1 (MSP1), a leucine-rich-repeat receptor kinase, plays an important role in anther development by limiting the number of sporocytes. *OsTDL1a* (a *TPD1*-like gene in rice) encodes a small protein that acts as a cofactor of MSP1 in the same regulatory pathway. In this study, we analyzed small RNA and mRNA changes in different stages of spikelets from wild-type rice, and from *msp1* and *ostdl1a* mutants. Analysis of the small RNA data identified miRNAs demonstrating differential abundances. miR2275 was depleted in the two rice mutants; this miRNA is specifically enriched in anthers and functions to trigger the production of 24-nt phased secondary siRNAs (phasiRNAs) from *PHAS* loci. We observed that the 24-nt phasiRNAs as well as their precursor *PHAS* mRNAs were also depleted in the two mutants. An analysis of co-expression identified three Argonaute-encoding genes (*OsAGO1d*, *OsAGO2b*, and *OsAGO18*) that accumulate transcripts coordinately with phasiRNAs, suggesting a functional relationship. By mRNA *in situ* analysis, we demonstrated a strong correlation between the spatiotemporal pattern of these *OsAGO* transcripts and phasiRNA accumulations.

## Highlight

Transcriptional analysis (mRNA and small RNA) of rice flower organs demonstrates correlations of 24-nt phased small RNAs with anther maturation and with three small RNA-binding Argonautes.

## Introduction

Rice (*Oryza sativa*), as a major crop, has been widely used as a monocot model species to explore the genetic basis of flower development in higher plants (Yoshida and Nagato, 2011). Rice anther development is one of the major topics studied in rice flower development, and changes in the cytological morphology in different developmental stages of rice anthers have been well-described (Zhang and Wilson, 2009; Zhang *et al.*, 2011). Tapetum and microsporocyte specification is a crucial event in male fertility, occurring at early stages of anther development in plants; a number of genes have been discovered as regulators of cell fate specification (Zhang and Yang, 2014). For example, EXCESS MICROSPOROCYTES 1 (EMS1, or EXTRA SPOROGENOUS CELLS, EXS), a member of the leucine-rich repeat receptor-like kinase (LRR-RLK) family, specifies tapetal identity and limits the number of pollen mother cells (PMCs) in Arabidopsis (Canales *et al.*, 2002; Zhao *et al.*, 2002). The small secreted protein, TAPETAL DETERMINANT 1 (TPD1), has been reported as a ligand of EMS1/EXS with a deterministic role in the cell fate of the tapetum (Jia *et al.*, 2008).

The EMS1/EXS ortholog in rice is MULTIPLE SPOROCYTE (MSP1) (Nonomura *et al.*, 2003), while the ligand protein TPD1 has two TPD1-like orthologs in rice, including OsTDL1A and OsTDL1B, among which OsTDL1A (also known as MICROSPORELESS2, MIL2) may interact with MSP1 (Zhao *et al.*, 2008; Hong *et al.*, 2012). Although *msp1* and *ostdl1a* mutants display defects in anther and ovule development, both show a phenotype of complete male sterility, while partially maintaining female fertility (Nonomura *et al.*, 2003; Hong *et al.*, 2012; Yang *et al.*, 2016). MSP1, as a receptor-like kinase in an upstream signaling pathway, affects many other downstream genes involved in rice anther development. For example, a loss of function of MSP1 will largely down-regulate the expression of other genes involved in rice anther development, such as *Undeveloped Tapetum1* (*UDT1*) and *Tapetum Degeneration Retardation* (*TDR*) (Jung *et al.*, 2005; Li *et al.*, 2006). Maize *MAC1* is the ortholog of *OsTDL1A*, having similar functions in limiting archesporial cell proliferation in maize anthers (Wang *et al.*, 2012). Therefore, the OsTDL1A-MSP1 pathway plays a central role in early stages of rice anther development to simultaneously specify the tapetum and limit the number of pollen mother cells (Zhang and Yang, 2014).

Small RNA pathways play roles in both flower development and gametogenesis in plants. Some conserved miRNAs appear to function similarly in flower development across different plant species, such as Arabidopsis, tomato, petunia, rice, and maize (Luo *et al.*, 2013). In rice, miR172 targets *APETALA2* (*AP2*) genes controlling inflorescence architecture and spikelet meristem identity (Zhu *et al.*, 2009; Lee and An, 2012). A number of rice *SQUAMOSA Promoter Binding Protein-Like* (*OsSPL*) genes, including *OsSPL14*, are targeted by miR156; this pathway has a role in flowering time, panicle architecture, grain yield, and other developmental phenotypes (Xie *et al.*, 2006; Jiao *et al.*, 2010; Miura *et al.*, 2010). Other miRNAs, such as miR159 and miR164, are also reported to be involved in rice floral development (Tsuji *et al.*, 2006; Adam *et al.*, 2011). In addition to miRNAs, *trans*-acting siRNAs (tasiRNAs), dependent on the activities of RNA-DEPENDENT RNA POLYMERASE 6 (RDR6) and DICER-LIKE 4 (DCL4), generated from non-coding transcripts such as *TAS3*, play roles in both vegetative and reproductive development in both rice and maize by targeting genes encoding auxin response factors (ARFs). For example, the rice mutant of *SHOOTLESS2* (*SHL2*), the ortholog of Arabidopsis *RDR6*, displays a severe phenotype of misregulation of adaxial-abaxial polarity patterning in both the lemma and anther (Toriba *et al.*, 2010), while the maize mutant *leafbladeless1 (lbl1*, a loss of function of an *SGS3* ortholog, *SUPPRESSOR OF GENE SILENCING 3*) is defective in tasiRNA biogenesis and shows a pleotropic phenotype, including sterile male inflorescences (Nogueira *et al.*, 2007). The RDR6-dependent small RNA biogenesis pathway not only produces tasiRNAs, but also yields two large populations of phased secondary siRNAs (phasiRNAs) in the reproductive tissues of monocots (reviewed in Fei *et al.*, 2013). Data have suggested that these 21- and 24-nt reproductive phasiRNAs, triggered by miR2118 and miR2275, respectively, may play crucial roles in microgametogenesis in maize, because the accumulation of phasiRNAs shows highly stage-specific patterns in maize anther development (Zhai *et al.*, 2015). In addition, the Argonaute (AGO) protein MEIOSIS ARRESTED AT LEPTOTENE1 (MEL1), previously demonstrated to be essential for sporogenesis in rice anthers (Nonomura *et al.*, 2007), has recently been shown to recruit 21-nt phasiRNAs (Komiya *et al.*, 2014).

In a recent study, we showed that the MSP1-OsTDL1A partners are master regulators of downstream transcription factors that are involved in plant anther development (Yang *et al.*, 2016). Therefore, we hypothesized that loss-of-function of MSP1-OsTDL1A may also cause great downstream changes in non-coding RNAs and small RNAs. Here, we systematically characterized changes in small RNA and mRNA, especially non-coding *PHAS* transcripts, across early developmental stages of rice spikelets in wild-type, *msp1* and *ostdl1a* backgrounds using deep sequencing data. We found comprehensive changes of miRNAs, phasiRNAs, and *PHAS* transcripts in early stages of rice spikelet development. Importantly, the reproductive phasiRNAs displayed stage-specific expression patterns during early stages of anther development, suggesting that the timing of phasiRNA biogenesis is crucial in rice microsporogenesis. Furthermore, phasiRNA and mRNA changes in different developmental stages and mutant backgrounds facilitated the identification of several rice AGOs, in addition to MEL1, that potentially load phasiRNAs.

## Materials and methods

### Plant materials and growth conditions

All the rice plants used in this study were in a genetic background of variety 9522, a *japonica* rice. The two male-sterile mutants, *ostdl1a* and *msp1-4*, are from a rice mutant library made by ^60^Co γ-ray radiation; the molecular details of these mutants are described in Yang *et al.* (2016). Plants were grown in the paddy field of Shanghai Jiao Tong University in China.

### Small RNA and RNA-seq library construction

For small RNA library construction, total RNA enriched for small RNA was extracted. The small RNA faction between 18 to 30 nt in length was collected by gel separation, then ligated to 5′ and 3′ adaptors and purified. These small RNAs were reverse transcribed by RT-PCR and finally amplified via PCR. For RNA-seq libraries, after the total RNA extraction and DNase I treatment, magnetic beads with oligo(dT) were used to isolate mRNA. Mixed with the fragmentation buffer, the mRNA was fragmented into short fragments. The cDNA was synthesized using the mRNA fragments as templates. Short fragments were purified for end repair and single nucleotide A (adenine) addition, ligated to adapters, and then the second strand was degraded using UNG (Uracil-N-Glycosylase). After agarose gel electrophoresis, the suitable fragments were selected for PCR amplification as templates. All the small RNA and RNA-seq libraries were sequenced on an Illumina HiSeq 2000 platform by BGI (BGI-Shenzhen, China).

### Small RNA data analysis

Small RNA sequencing data were preprocessed by removing adapters, and then mapped to the version 7.0 of the rice genome assembly from the Rice Genome Annotation Project Database (http://rice.plantbiology.msu.edu, accessed 23 September 2016) using the program Bowtie (Langmead *et al.*, 2009). Small RNA reads that were mapped to the tRNAs and rRNAs were filtered, and reads mapped to the rice genome from different libraries were normalized to reads per 5 million reads (RP5M) for comparisons. The Bioconductor (www.bioconductor.org) package ‘edgeR’ was used for small RNA differential analysis (*P*<0.05, FDR<0.05). Rice miRNA sequences were downloaded from miRBase (Release 21; http://www.mirbase.org/, accessed 23 September 2016) (Kozomara and Griffiths-Jones, 2014). The R (www.r-project.org, accessed 23 September 2016) package ‘pheatmap’ was used to represent the average abundance of miRNAs from three biological replicates. *PHAS* locus identification was performed using the same method described previously by Zhai *et al.* (2011). Briefly, small RNA sequencing data from different libraries were combined together to increase the sequencing depth for *PHAS* loci identification. A phasing score of 25 was used as a stringent cut-off, followed by a manual check to remove loci producing highly abundant small RNAs in other sizes, which are most likely degradation products from t/rRNAs. The overall phasiRNA abundance for each *PHAS* locus was calculated by summing up the normalized abundance of 21- or 24-nt small RNAs generated from each corresponding 21-*PHAS* and 24-*PHAS* locus.

### RNA-seq data analysis

Paired-end strand-specific RNA-seq reads (90bp × 2) were mapped to the rice genome sequences allowing no more than two mismatches using ‘Tophat’ (Trapnell *et al.*, 2009). The BAM files generated by ‘Tophat’ were sorted and indexed using ‘SAMtools’ (Li *et al.*, 2009), and then visualized via Integrative Genomics Viewer (IGV) (Robinson *et al.*, 2011). The program ‘Cufflinks’ (Trapnell *et al.*, 2012) was used for transcriptome assembly, differential analysis of gene expression, and calculation of the FPKM value (fragments per kilobase of transcript per million mapped reads). For differential analysis of gene expression, we used ‘q-value<0.01’ and ‘fold change > 2’ as cut-offs. Bar graphs and line charts representing FPKM values of gene expression were plotted using the Bioconductor package ‘cummeRbund’ (Trapnell *et al.*, 2012).

### Microarray data analysis

Gene lists were inputted into the webserver Rice Oligonucleotide Array Database (http://www.ricearray.org/, accessed 23 September 2016; Cao *et al.*, 2012). Specific public microarray datasets were selected to acquire the abundance values of each gene. The normalized gene expression values were further visualized as heatmaps using the R package ‘pheatmap’.

### *In situ* hybridizations

Freshly collected samples were fixed in formalin-acetic acid-alcohol (FAA) and dehydrated in a series of graded ethanol concentrations; these samples were then infiltrated with Histo-clear II, embedded in Paraplast Plus, and subsequently processed into 6-μm thick sections using a Leica RM2245 rotary microtome. Templates for RNA probe synthesis were amplified by PCR from the cDNA. Probes were transcribed *in vitro* under the T7 promoter with RNA polymerase, using the DIG RNA labeling kit (Roche). The RNA *in situ* hybridizations were carried out as described by Kouchi and Hata (1993) and Li *et al.* (2006). The forward and reverse RT-PCR primers were as follows: *OsAGO1d*, 5′-GCAATACCACCCACAAGGAC-3′ and 5′-GGTTCCAATACTCCCACTTCC-3′; *OsAGO18*, 5′-CAGTAT AACAGTACGGAACGC-3′ and 5′-TGTCATTACAACAAGTAG GAGG-3′.

### Accession numbers

RNA-seq and small RNA data are available from Genbank, under GEO accession number GSE77300.

## Results

### Comparative analysis of small RNAs in spikelets of wild-type and mutant rice

**Fig. 1.**
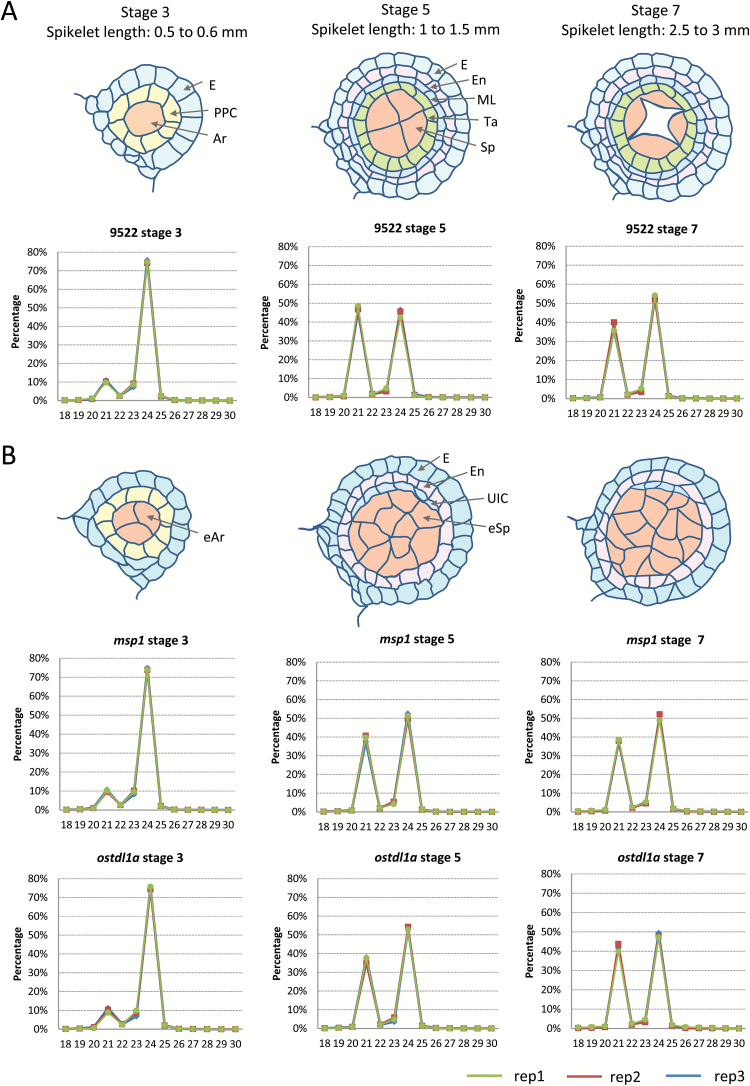
Small RNA size distribution in different developmental stages and backgrounds of rice spikelets. (A) Schematic representation of rice anther structures in different stages of spiklets of wild-type rice. Each layer of cells is indicated by an arrow: epidermis (E); primary parietal cell (PPC); archesporial cell (Ar); endothecium (En); middle layer (ML); tapetum (Ta); sporogenous cell (Sp). Small RNA size distributions in the different stages of wild-type rice spikelets are shown below. (B) Schematic representation of rice anther structures in different stages of spiklets of the *msp1* and *ostdl1a* mutants. Excessive archesporial cell (eAr); unknown identity cell (UIC); excessive sporogenous cell (eSp). Small RNA size distributions in the different stages of the mutant spikelets are shown below.

Small RNAs play crucial roles in mediating both transcription and translation, with different classes distinguishable by their distinct biogenesis pathways (Axtell, 2013). The recent discovery of reproductive phasiRNAs in monocots indicates that this special class of small RNAs may be important for male reproduction, although the underlying mechanism remains to be elucidated (Johnson *et al.*, 2009; Song *et al.*, 2012; Zhai *et al.*, 2015). To assess small RNA and mRNA changes across different stages of rice spikelet development and to understand how they are impacted by perturbation of the OsTDL1A-MSP1 pathway, we prepared small RNA and RNA-seq libraries from spikelets of wild-type rice cultivar 9522, and the mutants *msp1-4* (‘*msp1*’ hereafter) and *ostdl1a* [library information is listed in Supplementary Table S1 at *JXB* online; mutant information is described in Yang *et al.* (2016), and is also shown in Supplementary Fig. S1]. We performed three biological replicates for each genotype and stage. The lengths of rice spikelets correspond to different anther developmental stages (Fig. 1). Specifically, stage 3 (0.15–0.2mm), stage 5 (0.25–0.3mm), and stage 7 (0.4–0.45mm) of rice anthers correspond to 0.5–0.6mm, 1.0–1.5mm, and 2.5–3.0mm rice spikelets, respectively (Zhang *et al.*, 2011) (Fig. 1A); hereafter, we will refer to these sizes of rice spikelets as stage 3, stage 5, and stage 7 spikelets, respectively. Samples were collected at these three stages because MSP1 and OsTDL1A mainly function at early stages (stage 3 to stage 5) of rice anther development (Yang *et al.*, 2016), while stage 5–7 is an important stage at which meiocytes start meiosis (Zhang *et al.*, 2011).

Sequencing reads of small RNAs were aligned to the rice genome and normalized to 5 million (5M), and the distribution of lengths in different stages was analyzed. The three replicates were nearly identical (Fig.1). The 24-nt small RNAs account for ~75% of all small RNAs at stage 3 in wild-type rice. Interestingly, with the development of spikelets, there was a shift in the predominant size class, with 21-nt small RNAs the largest proportion (nearly 50%) in the profile at stage 5. In stage 7, the proportion (~55%) of 24-nt small RNAs was once again larger than the 21-nt counterpart (<40%), but was far smaller than that in stage 3. The fluctuation of small RNA percentages in different sizes that accompanied spikelet development may represent shifts in small RNA biogenesis and transcriptome changes in rice reproductive tissues, consistent with prior reports in maize (Zhai *et al.*, 2015). Similar to the wild-type spikelets, in the two mutants changes in small RNA size proportions occurred more significantly in the transition from stage 3 to stage 5 than from stage 5 to stage 7. The only apparent difference was a reduction in the proportion of 21-nt small RNAs in the mutants in stage 5 spikelets (Fig. 1B). To investigate this difference, and others less readily apparent, we checked the levels of small RNAs that are often 21 nt in length, miRNAs and phasiRNAs.

### miRNA expression patterns in different developmental stages of rice spikelets

A number of miRNAs have characterized roles in plant development, targeting several families of transcription factors or other development-related genes (Jones-Rhoades *et al.*, 2006; Chen, 2009). miRNAs, such as miR156 and miR172, have been proven to control flower development at a post-transcriptional level in both Arabidopsis and rice (Aukerman and Sakai, 2003; Chen, 2004; Xie *et al.*, 2006; Wu *et al.*, 2009*a*; Zhu *et al.*, 2009; Jiao *et al.*, 2010; Lee and An, 2012). Therefore, the expression patterns of miRNAs are important to our understanding of rice spikelet development.

**Fig. 2.**
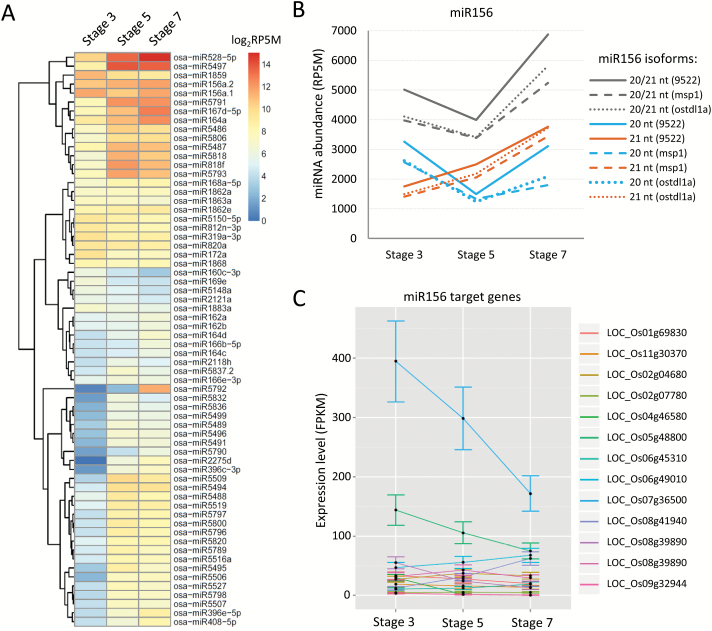
miRNA expression in different developmental stages of spikelets in wild-type rice cultivar 9522. (A) Differentially expressed miRNAs in different developmental stages of spikelets. Only miRNAs with abundance greater than 50 RP5M are included in the heatmap. (B) 20-nt miR156 and 21-nt isoforms display distinct expression patterns; levels indicated as ‘20/21’ show the sum of abundance of the 20 and 21 nt isoforms. (C) Expression levels of miR156 target genes in different stages of rice spikelet development.

Genome-wide differential analysis of miRNA expression was performed in wild-type rice across different stages of spikelet development (see Supplementary Tables S2 and S3). From stage 3 to stage 5, >60 miRNAs increased significantly, while only 18 miRNAs decreased during the same time period; from stage 5 to stage 7, the numbers of miRNAs with significantly different levels (up or down) were both fewer than 20 (Fig. 2A; Table 1). Comparing different stages in the *msp1* and *ostdl1a* mutant backgrounds, similar sets (both qualitatively and quantitatively) of differentially accumulating miRNAs were observed, suggesting that the male sterile phenotype of these mutants has a limited impact on miRNA levels, relative to the wild-type. This was further confirmed by the observation that very few miRNAs were identified as significantly different in their levels when comparing the two mutants with the wild-type (Table 1). Interestingly, among the few impacted miRNAs in the two mutants, miR2275, which triggers 24-nt phasiRNA production, was totally abolished (Supplementary Table S3, and see below).

**Table 1.** Numbers of differentially abundant miRNAs, identified by pairwise comparisons of rice spikelets in different stages and backgrounds.

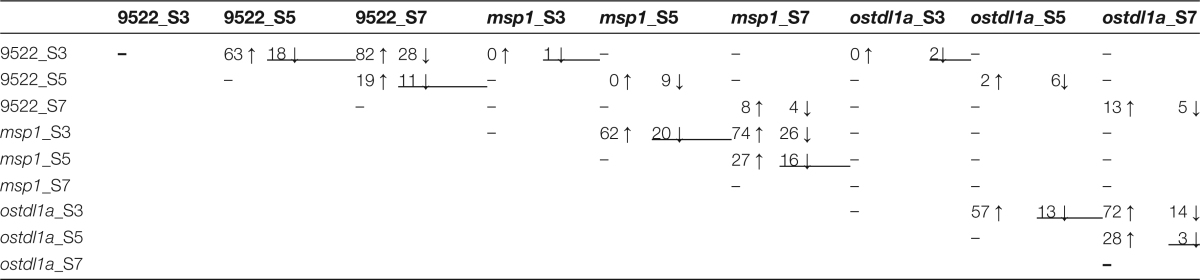

Numbers of significantly up- and down-regulated miRNAs, as indicated by the arrows (*P*<0.05, FDR<0.05). miRNAs are from the miRBase release 21. ‘S3’, ‘S5’, and ‘S7’ refer to stages 3, 5, and 7, respectively.

Among the miRNAs differentially expressed in wild-type rice across the developmental stages, the level of miR164 showed a ~7-fold increase from stage 3 to stage 7, whereas miR172 showed a dramatic decline of ~4-fold from stage 3 to stage 5, then remained relatively steady to stage 7 (see Supplementary Fig. S2A). Prior work using PARE/degradome data confirmed the targets of a number of miRNAs in rice (Li *et al.*, 2010; Zhou *et al.*, 2010). From RNA-seq data, we obtained the expression levels of both miR164 and miR172 target genes. Transcript levels of the most abundant miR164 target (LOC_Os12g05260) decreased from 180 FPKM (stage 3) to 125 FPKM (stage 5), and the expression level increased slightly in stage 7 compared to stage 5, and a similar trend was observed for the target gene LOC_Os06g23650 (Supplementary Fig. S2B); these were the only two targets that showed an inverse relationship with miR164 abundance. Three miR172 targets showed an inverse correlation with miR172 levels (Supplementary Fig. S2C). miR156 showed an interesting pattern across the developmental stages of rice spikelets: a 21-nt miR156 isoform increased gradually from stage 3 to stage 7, while the 20-nt miR156 firstly decreased from stage 3 to stage 5, and then increased at stage 7 (Fig. 2B). The underlying mechanism is unknown, but it is possible that these two isoforms of miR156 are generated from different *MIR156* genes, and are differentially regulated with distinct targets or cellular expression patterns, exerting distinct patterns of control on the expression of *OsSPLs*. Therefore, studies of individual *MIR156* genes would be important in the future to demonstrate how the miR156 family members differentially control spikelet or panicle development. Similar to miR164 and miR172, a subset of miR156 targets showed an inverse correlation with miR156 (Fig. 2C). Overall, the abundance levels of miRNA target transcripts in the two mutants were quite similar to those in wild-type, indicating that mutations in the OsTDL1A-MSP1 pathway had a limited impact on rice miRNAs and their targets in spikelets (Table 1; Supplementary Fig. S2B–D). Among the miRNA targets validated from prior work, only a subset had the expected inverse correlation with miRNA levels; thus, other factors may impact target transcript levels, including both transcriptional (e.g. transcription factors and epigenetic regulation) and post-transcriptional (e.g. mRNA turnover or sequestration) factors. Taken together, these data indicate that miRNAs together with their target genes are dynamically modulated during early stages of rice spikelet development, and are largely independent of the OsTDL1A-MSP1 pathway.

### Timing of reproductive phasiRNA biogenesis

**Fig. 3.**
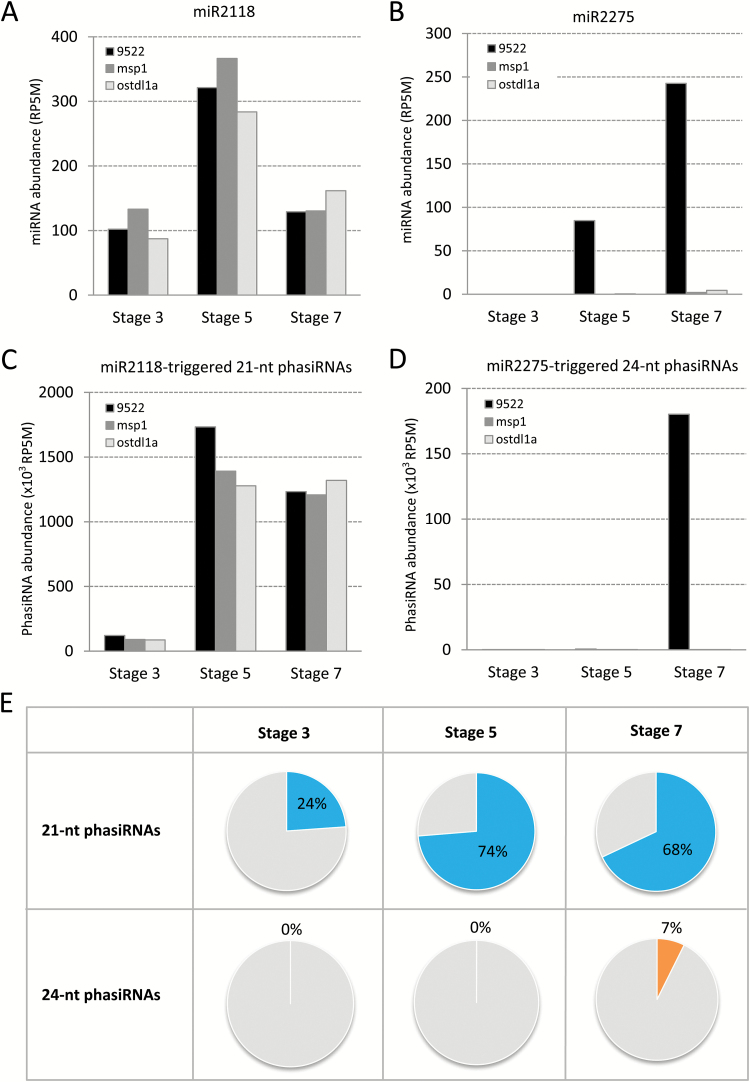
miR2118, miR2275, and phasiRNA abundances in rice spikelets. Levels of miR2118 (A), miR2275 (B), miR2118-triggered 21-nt phasiRNAs (C), and miR2275-triggered 24-nt phasiRNAs (D) in different stages and backgrounds of rice spikelets. (E) The percentage of 21-nt (top, blue slices with percentage numbers) and 24-nt phasiRNAs (bottom, orange slices with percentage numbers) out of the total population of 21-nt and 24-nt genome-matched small RNAs in wild-type rice spikelets.

PhasiRNAs, in addition to miRNAs, represent another class of small RNAs of great interest, because two populations are exclusively abundant in the reproductive tissues of monocots (Arikit *et al.*, 2013). More specifically, miR2118 and miR2275 are triggers of 21-nt and 24-nt reproductive phasiRNAs, respectively, in both rice and maize (Song *et al.*, 2012; Zhai *et al.*, 2015). We checked the abundance levels of both miRNAs in different stages and backgrounds; miR2118 abundance peaked at stage 5 in wild-type spikelet libraries, reaching 320 reads per 5 million reads (RP5M), and then dropped to 130 RP5M at stage 7 (Fig. 3A). Similar abundances were observed in *msp1* and *ostdl1a* mutants, indicating that miR2118 is not impacted in both mutants, possibly explained by miR2118 accumulation in the epidermis of anthers (Zhai *et al.*, 2015), a cell layer apparently not defective in the *msp1* and *ostdl1a* mutants or in the *mac1* maize mutant. In contrast to miR2118, in wild-type spikelets, miR2275 was not expressed in stage 3, and then increased in stage 5 (~85 RP5M) and stage 7 (~240 RP5M) (Fig. 3B); miR2275, as mentioned above, is essentially absent in both *msp1* and *ostdl1a* mutants, at all stages.

We calculated both 21- and 24-nt phasiRNA abundances to see how phasiRNAs change during rice spikelet development and whether they are affected in the two mutants. Phasing analysis resulted in 1843 21-*PHAS* loci and 50 24-*PHAS* loci (see Supplementary Table S4). By summing phasiRNA abundances from each *PHAS* locus, we obtained the overall phasiRNA abundances for both 21- and 24-nt phasiRNAs. In wild-type rice spikelets, the overall abundance of 21-nt phasiRNAs was ~120 000 RP5M in stage 3, increasing by >10-fold in stage 5, and then decreasing by <30% in stage 7 (Fig. 3C). Compared to the wild-type, 21-nt phasiRNAs had a very similar pattern in the two mutants, although with a slightly lower total abundance in stage 5. The 24-nt counterparts had only a few hundred reads in stage 3 and stage 5, but then increased to more than 180 000 RP5M in stage 7 of the wild-type spikelets (Fig. 3D). Consistent with the observation that miR2275 is absent in *msp1* and *ostdl1a* mutants at all stages, 24-nt phasiRNAs were also diminished in both mutants. Considering the developmental defect in these mutants is largely in the anthers and not other tissues in the spikelets (Yang *et al.*, 2016), we infer that the loss of 24-nt phasiRNAs is due to the defective anther development. Furthermore, as in maize (Zhai *et al.*, 2015), rice 21-nt phasiRNAs initiated at an early stage in anther development, the 24-nt phasiRNAs appeared later, coincident with or just before meiosis, and the absence of miR2275 and 24-nt phasiRNAs in *msp1* and *ostdl1a* mutants is similar to maize *mac1* (the ortholog of *ostdl1a* in maize).

We next calculated the proportion of 21- and 24-nt phasiRNAs in the entire genome-matched populations of the 21- and 24-nt small RNAs in rice spikelets (Fig. 3E). We found that 21-nt phasiRNAs accounted for 24% of total 21-nt siRNAs in wild-type rice spikelets, increased to a remarkable 74% of the total in stage 5, and then reduced slightly to 68% at stage 7. This compares to 60% of all 21-mers at the peak in maize (Zhai *et al.*, 2015); this higher proportion in rice spikelets versus isolated maize anthers may reflect the fact that there are ~4-fold as many genomic loci generating 21-nt phasiRNAs in rice compared to maize. The 24-nt phasiRNAs were almost absent at stages 3 and 5, followed by a substantial increase to 7% of the total at stage 7 (Fig. 3E). This compares to 64% of the total 24-mers in isolated maize anthers (Zhai *et al.*, 2015); this much lower proportion in rice may reflect the fact that there are fewer loci, or that the peak abundance of 24-nt phasiRNAs is later than stage 7. Overall, these results reveal that both miRNA triggers and phasiRNAs are largely up-regulated at specific stages in rice anthers.

### Interconnected *PHAS* locus transcription and phasiRNA bursts

**Fig. 4.**
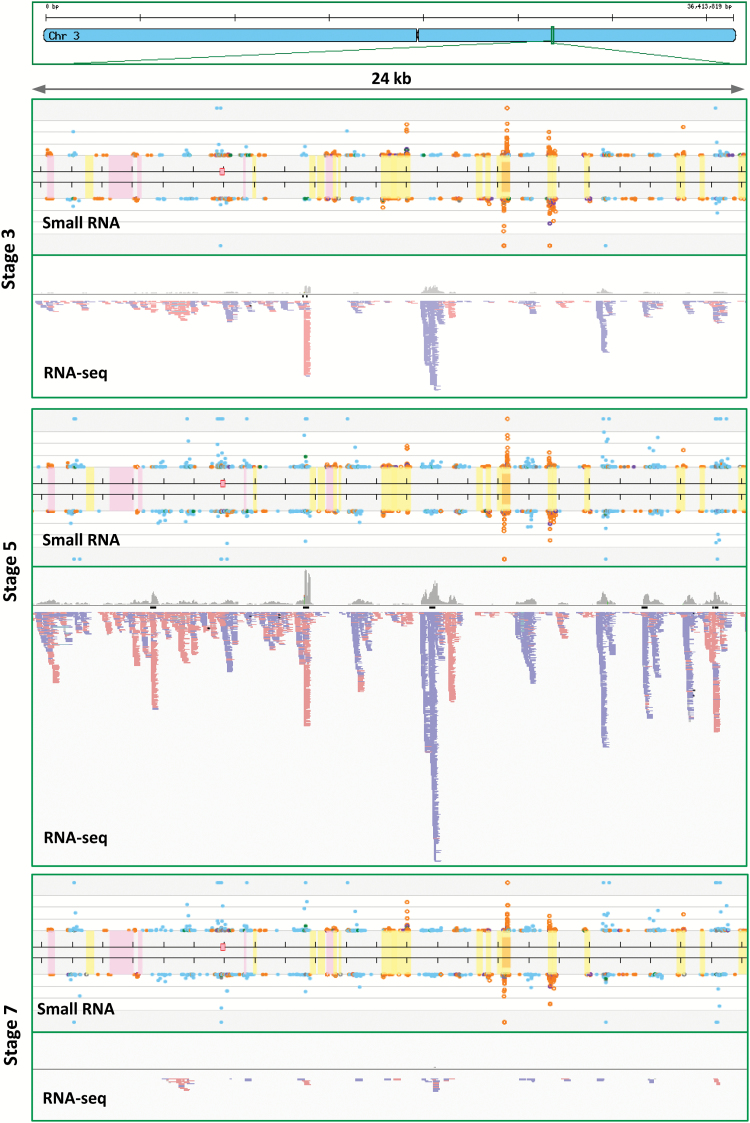
21-nt *PHAS* precursor transcripts peak coincidentally with their phasiRNA products. We examined a randomly selected 21*-PHAS* locus on rice chromosome 3 in wild-type (cultivar 9522) rice to assess the peak of abundance relative to the 21-phasiRNAs that peak at stage 5. Because *PHAS* loci are highly clustered in the rice genome, we selected a region of ~24kb as an example; in this case, the *PHAS* loci are interlaced with repetitive sequences. Each dot is a small RNA; light blue represent 21-nt sRNAs, green represent 22-nt, and orange represent 24-nt. Yellow shaded regions are predicted DNA transposons; pink shaded regions are predicted retrotransposons; orange shaded regions are inverted repeats. The small pink box is an annotated miRNA. The phasiRNA loci are essentially the distinct blocks of 21-nt sRNAs (light blue dots). The strand-specific RNA-seq data is represented as an IGV screenshot; blue bars are top-strand reads, and red bars are bottom-strand reads. There was a paucity of RNA-seq reads from these *PHAS* loci data in stage 7 at this cluster of loci, whereas 21-phasiRNAs were still abundant at stage 7.

We next examined the phasiRNA precursors (*PHAS* transcripts) in the RNA-seq data to assess the correlation of phasiRNA and *PHAS* mRNA levels. As many 21-*PHAS* loci are found in large clusters in the rice genome (Johnson *et al.*, 2009) (see Supplementary Table S4), we selected a representative cluster on chromosome 3 for this analysis. Our earlier observations in wild-type rice showed a stage 5 peak for 21-nt phasiRNAs, which reduced slightly at stage 7 (Fig. 3C); in the RNA-seq data at stage 3 and stage 5, 21-*PHAS* transcripts levels were generally consistent with phasiRNA production; however, at stage 7, 21-*PHAS* transcripts levels were very low (Fig. 4) while phasiRNA levels were still high. This is an indication that 21-nt phasiRNAs persist longer than their precursor transcripts, possibly for a prolonged role in later stages of spikelet development. As for *msp1* and *ostdl1a* mutants, levels of neither 21-nt phasiRNAs nor 21-*PHAS* transcripts were impacted (stage 5 for both mutants is shown in Supplementary Fig. S3; stage 7 data are not shown but were not appreciably different from the wild-type). Consistent with the precursor molecules consisting of a polyadenylated mRNA paired with an RDR6-derived antisense strand, the strand-specific RNA-seq reads were mapped to only one strand at a given *PHAS* locus (Fig. 4). An examination of the RNA-seq data for 24-*PHAS* loci showed a strong boost in transcript levels from stage 5 to stage 7 in wild-type rice anthers (Supplementary Fig. S4A). However, unlike 21-*PHAS* loci, both 24-nt phasiRNAs and 24-*PHAS* mRNAs were absent in the RNA-seq data from *msp1* and *ostdl1a* mutants (Supplementary Fig. S4A, B). In summary, sequencing data revealed the developmental modulation of phasiRNA biogenesis in rice anthers, with 24-phasiRNAs and their precursors disrupted in the *msp1* and *ostdl1a* mutants, reflecting an intimate association of these transcripts with anther development and microsporogenesis in rice.

### Identification of specialized Argonautes that load reproductive phasiRNAs

**Fig. 5.**
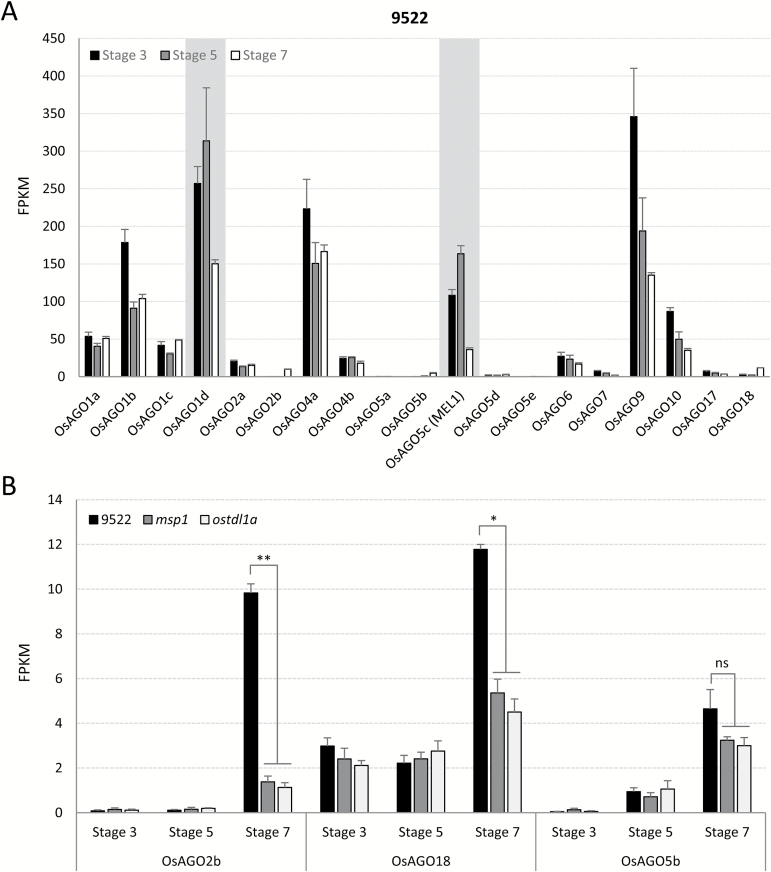
Abundance of *OsAGO* transcripts in different stages and backgrounds of anther development. (A) mRNA levels of all nineteen *OsAGOs* in wild-type rice. *OsAGO1d* and *MEL1* are highlighted because only these two *AGO* transcripts peaked at stage 5. (B) *OsAGO2b*, *OsAGO18*, and *OsAGO5b* displayed substantial up-regulation at stage 7 compared to stages 3 and 5. *OsAGO2b* and *OsAGO18*, but not *OsAGO5b*, is defective in the *msp1* and *ostdl1a* mutants at stage 7. Significant differences (Student’s *t*-test) are indicated: *P<0.01 and **P<0.05; ‘ns’ indicates no significant difference.

Argonautes (AGOs) are core effector proteins in small RNA-mediated silencing pathways. Different small RNAs are preferentially recruited into specific AGOs, mainly determined by the 5′-terminal nucleotide of the small RNA (Mi *et al.*, 2008). The ten AGO proteins of Arabidopsis are reasonably well studied, but the functional roles of the ~17 to 19 AGOs in grass genomes are less well described (Zhang *et al.*, 2015). The naming for OsAGOs in this study is consistent with the description by Zhang *et al.* (2015). Moreover, it is still unclear which AGOs recruit the abundant reproductive phasiRNAs of grasses. A recent study in rice showed that the germline-specific AGO protein MEL1 associates with 21-nt phasiRNAs that have 5′-terminal cytosine (Komiya *et al.*, 2014). In addition, the AGO(s) that recruits 24-nt phasiRNAs is still unknown, although *ZmAGO18b* is enriched in tapetum and germ cells in maize anthers (Zhai *et al.*, 2014), where 24-nt phasiRNAs accumulate (Zhai *et al.*, 2015). Considering the dramatic changes of phasiRNA abundances across different stages of rice spikelet development, we hypothesized that AGO proteins that recruit phasiRNAs may show a gene expression pattern correlated with phasiRNA abundances. Therefore, we examined the expression of all AGOs in rice from stages 3 to 7 (Fig. 5A). We found that *MEL1* peaked at stage 5, the same stage as the peak of accumulation of 21-nt phasiRNAs; therefore, *MEL1* expression was indeed correlated with phasiRNA production. In the RNA-seq data, *MEL1* levels were slightly higher in both mutants (see Supplementary Fig. S5), perhaps because of the excessive number of sporocytes, where *MEL1* is expressed, in mutants (Nonomura *et al.*, 2007). Intriguingly, we found that *OsAGO1d* showed the same pattern as *MEL1*, suggesting a possible functional connection between the OsAGO1d protein and reproductive phasiRNAs (Fig. 5A). Similar to *MEL1*, *OsAGO1d* expression was barely impacted in *msp1* and *ostdl1a* mutants (Supplementary Fig. S5).

We speculated that AGOs that accumulate in stage 7 could function with 24-nt phasiRNAs. Gene expression analysis showed that only three *OsAGO*s, namely *OsAGO2b*, *OsAGO5b*, and *OsAGO18*, displayed a substantial up-regulation at stage 7 compared to stages 3 and 5 (Fig. 5B). Since 24-nt phasiRNA accumulation was deficient in *msp1* and *ostdl1a* mutants, the result of defects in cell layers important for their biogenesis, it is possible that the *OsAGO*s that load 24-nt phasiRNAs are similarly impacted in both mutants. We found that the levels of *OsAGO2b* and *OsAGO18*, but not *OsAGO5b*, were partially reduced in the *msp1* and *ostdl1a* mutants (Fig. 5B), suggesting that OsAGO2b and OsAGO18 are candidates for roles with 24-nt phasiRNAs. To further confirm their specificity to anthers, we checked their expression patterns using published rice anther microarray datasets. Consistent with our RNA-seq data, *OsAGO2b* and *OsAGO18*, but not *OsAGO5b*, showed a meiosis-specific expression pattern in rice anthers (see Supplementary Fig. S6).

### Temporal-spatial expression of phasiRNA-associated AGOs revealed by *in situ* hybridizations

**Fig. 6.**
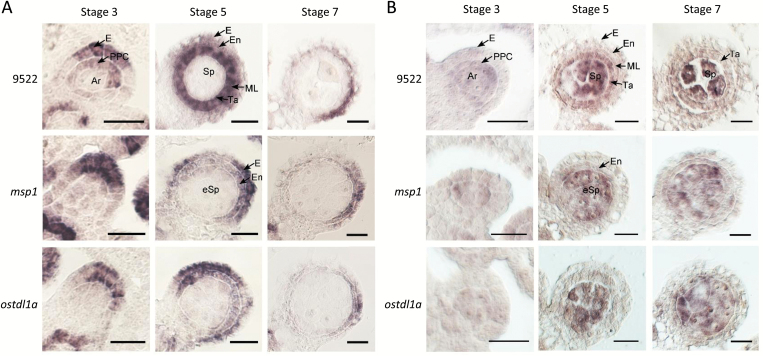
RNA *in situ* hybridization of *OsAGO1d* and *OsAGO18* in different stages and backgrounds of rice anthers. Expression patterns of *OsAGO1d* (A) and *OsAGO18* (B), performed in stages 3, 5, and 7 of anthers, from either wild-type cultivar 9522 or the *msp1* or *ostdl1a* mutants, as indicated. Each image shows one anther lobe. Cell layers are labeled as in Fig. 1. Scale bars indicate 20 μm.

To connect the temporal specificity of transcript accumulation with spatial patterns, we performed *in situ* hybridizations or examined published images for selected rice *AGO* genes. Published images for *OsAGO2b* demonstrate its accumulation in sporocytes and wall layers of rice anthers at pre-meiotic stages, and in later meiosis stage the transcript is restricted predominantly to the tapetum layer and microspores (Deveshwar *et al.*, 2011). We performed *in situ* hybridizations in rice anthers to examine the transcript accumulation patterns of *OsAGO1d* and *OsAGO18* (Fig. 6). These results showed that *OsAGO1d* accumulates highly in the distal epidermis and primary parietal cells of the anther lobe at stage 3. This *OsAGO1d* pattern is reminiscent of miR2118, which accumulates in the distal cells of the epidermis in maize anthers (Zhai *et al.*, 2015). In stage 5, *OsAGO1d* accumulates highly in the middle layer and the tapetal layer, which are both differentiated from the primary parietal cells. In stage 7, the level of *OsAGO1d* is much lower than that of stage 5 (Fig. 6A). As for *OsAGO18*, its abundance was enriched in tapetal and sporogenous cells at stages 5 and 7 (Fig. 6B). Therefore, the patterns of *OsAGO1d* and *OsAGO18* across different developmental stages of rice anthers are consistent with the results of RNA-seq data from rice spikelets, and these *OsAGO* transcripts are highly correlated both spatially and temporally with reproductive phasiRNAs (comparing our *in situs* with those of the phasiRNAs in maize anthers in Zhai *et al.*, 2015). Taken together, these analyses suggest a possible functional connection between grass reproductive phasiRNAs and the three AGO proteins encoded by *OsAGO1d*, *OsAGO2b*, and *OsAGO18*.

## Discussion

Like most complex developmental changes, rice spikelet development entails widespread changes in mRNAs and small RNAs. Similar data for mutants in *MSP1* and *OsTDL1A*, which encode interacting proteins with crucial roles in initiating early stage reproductive development, demonstrate the impact of disordered cell specification in rice reproductive tissues (Yang *et al.*, 2016).

miRNAs play important roles in plant development. We identified miRNAs with differential accumulation patterns during rice spikelet development, including conserved miRNAs (miR156, miR172, and miR164). Among these, the role of miR164 in development is not well studied, although it accumulates in spikelet and floral meristems (Adam *et al.*, 2011). While variation in miRNA levels in different stages of rice spikelets suggests that miRNAs are active, one interesting observation from the *msp1* and *ostdl1a* mutants is that levels of most miRNAs and their targets were largely not impacted in the mutants, despite the block in development (Fig. 2B, C; Supplementary Fig. S2). This suggests that these miRNA-involved gene silencing pathways are genetically independent or upstream of the OsTDL1A-MSP1 pathway.

We also assessed phasiRNA production in rice spikelets. Two classes of phasiRNAs have distinct accumulation patterns in grass anthers (Zhai *et al.*, 2015); as in maize, we found that rice phasiRNAs peak at specific stages during spikelet development. We showed that miR2118, the trigger of 21-nt phasiRNAs, accumulates to the highest level at stage 5 and drops severely in stage 7, while 21-nt phasiRNAs are relatively slightly retarded, peaking at stage 5 but decreasing only slightly at stage 7. As in maize, miR2275 peaks later (stage 7), at the stage at which 24-nt phasiRNAs reached the highest abundance that we measured. In the *msp1* and *ostdl1a* mutants, 24- but not 21-nt phasiRNAs were depleted – consistent with data from the maize mutant *mac1* (the ortholog of rice OsTDL1A) (Wang *et al.*, 2012). Therefore, the timing of phasiRNA biogenesis is conserved in rice and maize, two Poaceae evolutionarily separated by ~50 million years (Wolfe *et al.*, 1989).

AGO proteins are key catalytic components that associate with small RNAs, and different AGOs function as either RNA binders/slicers or chromatin modifiers by loading different classes of small RNAs (Waterhouse, 2016). Therefore, to know which AGOs load these reproductive phasiRNAs would greatly help elucidate their functions. Considering that in the mutants 24-nt phasiRNAs were impacted but miRNAs were largely not, we may be able to infer AGOs with roles in phasiRNA function. Compared to 10 AGOs in Arabidopsis, the rice and maize genomes encode more, 19 and 17 respectively (Zhang *et al.*, 2015). *AGO5* expression in Arabidopsis is specific to somatic ovule tissues, with a role in megagametogenesis (Tucker *et al.*, 2012). A potentially conserved function of AGO5 in plant gametogenesis has been shown in rice, as the AGO5 relative MEL1 (OsAGO5c) binds 21-nt reproductive phasiRNAs with 5′ C (Komiya *et al.*, 2014). AGO1 in rice has four homologs (OsAGO1a, OsAGO1b, OsAGO1c, and OsAGO1d); OsAGO1a/b/c predominantly recruit miRNAs and other small RNAs with 5′-terminal uridine (Wu *et al.*, 2009*b*). Our RNA-seq data showed that rice *OsAGO1d* accumulates in spikelets, synchronous with *MEL1*, making *OsAGO1d* a strong candidate for further functional analysis. In rice, *OsAGO18* is induced upon viral infection, and has been shown to confer resistance to viruses by sequestering miR168, suppressing *OsAGO1* expression (Wu *et al.*, 2015). Maize has two homologs of AGO18; ZmAGO18b is specific to the tapetum and germ cells (Zhai *et al.*, 2014). Our data showed that *OsAGO18* and *OsAGO2b* transcripts increase substantially at stage 7, coincident with 24-nt phasiRNA accumulation. Moreover, a recent study on Arabidopsis AGO3 showed that this poorly characterized Argonaute mediates RdDM by binding 24-nt siRNAs (Zhang *et al.*, 2016). Phylogenetic analysis showed that Arabidopsis AGO3 and rice OsAGO2b are close to each other (Zhang *et al.*, 2015), suggesting that OsAGO2b could also recruit 24-nt small RNAs, such as the reproductive phasiRNAs, to regulate epigenetic modifications. Furthermore, *in situ* hybridization results for *OsAGO1d*, *OsAGO18*, and *OsAGO2b* showed that these *OsAGO*s have distinct expression patterns during rice anther development, suggesting that they may associate with either 21- or 24-nt phasiRNAs in a stage-specific manner. In summary, our results suggest a functional relevance between OsAGO1d, OsAGO18, and OsAGO2b and grass reproductive phasiRNAs.

What might be the function of these AGO proteins loaded with phasiRNAs? Prior work on MEL1 (associated with 21-nt phasiRNAs) suggests a role in histone modifications; histone H3 lysine 9 dimethylation (H3K9me2) is decreased in the *mel1* mutant (Nonomura *et al.*, 2007). Yet the reduced H3K9me2 in *mel1* could be an indirect effect of other epigenetic changes, as many chromatin modifications in plants and other organisms are intricately linked (Castel and Martienssen, 2013). Considering the lack of sequence complementarity of phasiRNAs and other regions in the genome (Zhai *et al.*, 2015), it is possible that reproductive phasiRNAs act primarily in *cis* or impact *cis*-adjacent regions by a spreading mechanism. Indeed, a recent study on maize reported that both 21- and 24-*PHAS* loci showed higher levels of CHH methylation in meiocytes than other tissues, such as seedlings (Dukowic-Schulze *et al.*, 2016). This finding suggests that reproductive phasiRNAs may play an important role in chromatin remodeling in *cis* around the stage of meiosis. Overall, the coordinated accumulation of 21- and 24-nt phasiRNAs and several *AGO* transcripts during rice reproductive development suggests that more detailed and comprehensive analyses of DNA methylation and histone modifications are needed, particularly when coupled with mutants in phasiRNA pathways.

## Supplementary data

Supplementary data are available at *JXB* online.

Figure S1. *MSP1* and *OsTDL1A* transcript levels in stage 3 of rice spikelets in the wild-type cultivar 9522 and two mutants.

Figure S2. miRNA and target transcript levels in different stages and backgrounds of rice spikelets.

Figure S3. 21-nt phasiRNAs and precursor transcripts were unaffected in both *msp1* and *ostdl1a* mutants at stage 5.

Figure S4. 24-nt phasiRNAs are strongly impacted in stage 7 spikelets of the two rice mutants.

Figure S5. mRNA levels of *MEL1* and *OsAGO1d* in different stages and backgrounds of rice spikelets.

Figure S6. Expression of AGOs in different tissues and developmental stages of rice anthers in public microarray datasets.

Table S1. Summary information for small RNA and RNA-seq libraries prepared in this study.

Table S2. miRNA levels in different libraries.

Table S3. Differentially expressed miRNAs in different stages and backgrounds of rice spikelet development.

Table S4. 21- and 24-nt phasiRNA abundance from each *PHAS* locus.

Supplementary Data
