# Supplementary material for: Dynamic changes of small RNAs in rice spikelet development reveal specialized reproductive phasiRNA pathways
Source: J Exp Bot. 2016 Oct 4;67(21):6037–49. doi: 10.1093/jxb/erw361 (PMC5100018; doi:10.1093/jxb/erw361)
Supplement: Supplementary Data [file supp_67_21_6037__index.html]

Dynamic changes of small RNAs in rice spikelet development reveal specialized reproductive phasiRNA pathways — Dynamic changes of small RNAs in rice spikelet development reveal specialized reproductive phasiRNA pathways — Supplementary Data 

# Dynamic changes of small RNAs in rice spikelet development reveal specialized reproductive phasiRNA pathways

## Supplementary Data

Data files

- supplementary\_figures\_S1\_S6.pdf - Supplementary Data
- supplementary\_table\_S1.xlsx - Supplementary Data
- supplementary\_table\_S2.xlsx - Supplementary Data
- supplementary\_table\_S3.xlsx - Supplementary Data
- supplementary\_table\_S4.xlsx - Supplementary Data
