# Supplementary material for: Dynamic changes of small RNAs in rice spikelet development reveal specialized reproductive phasiRNA pathways
Source: J Exp Bot. 2016 Oct 4;67(21):6037–49. doi: 10.1093/jxb/erw361 (PMC5100018; doi:10.1093/jxb/erw361)
Supplement: Supplementary Data [file supp_erw361_supplementary_figures_S1_S6.pdf]

A

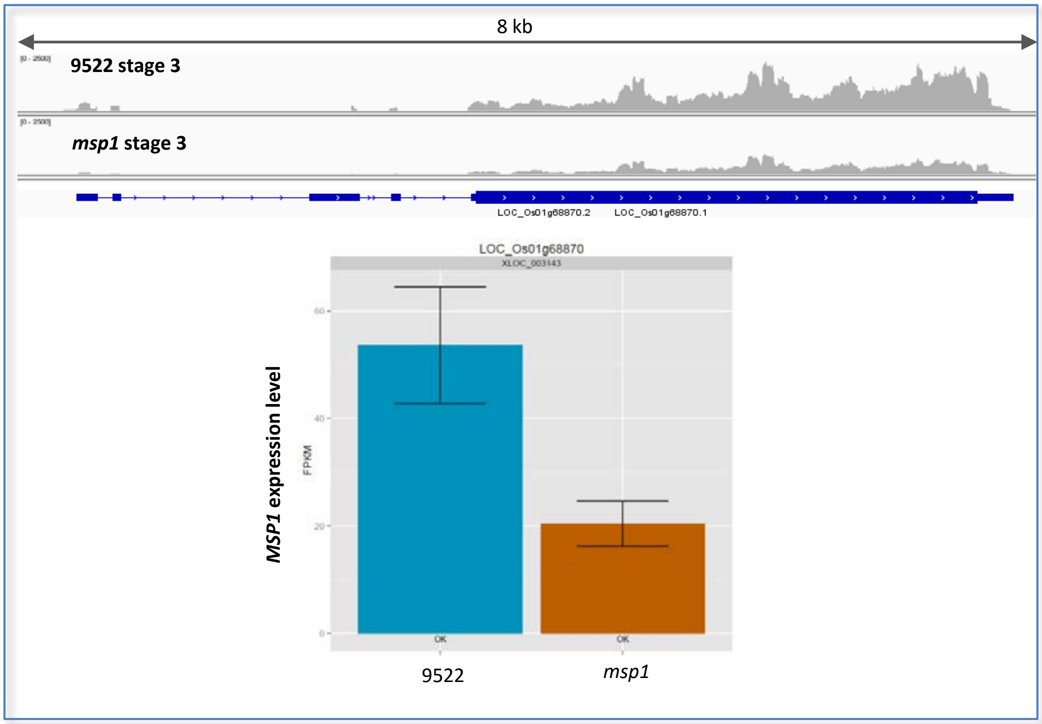

B

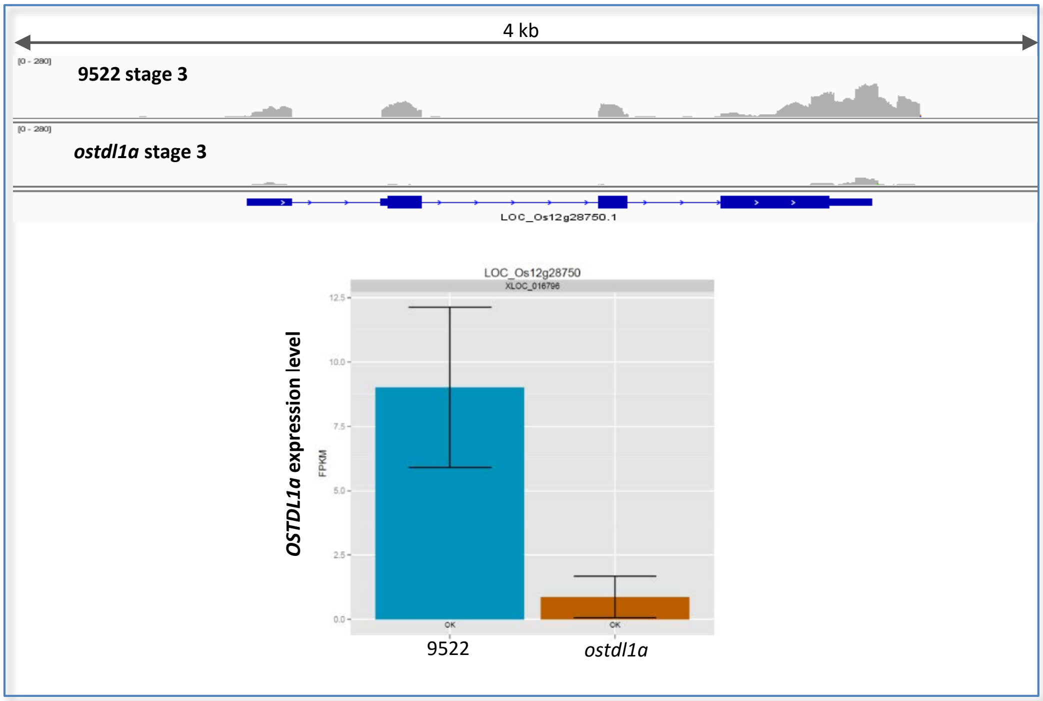

Figure S1. ***MSP1*** **and** ***OsTDL1a*** **transcript levels in stage 3 of rice spikelets in wild type 9522 and two mutants.** Shown are IGV screenshots (upper portion of each panel, with a scale bar in kilobases at the very top) showing the annotated rice gene in blue and the combined or merged set of reads from three replicate RNA-seq libraries mapped to each locus, for either the wildtype (upper grey plot) or mutant (lower grey plot). The transcript levels of *MSP1* (A) and *OsTDL1A* (B) were substantially reduced in the respective mutants. In each panel, the lower box plot indicates the FPKM levels derived from the data in the IGV plots.

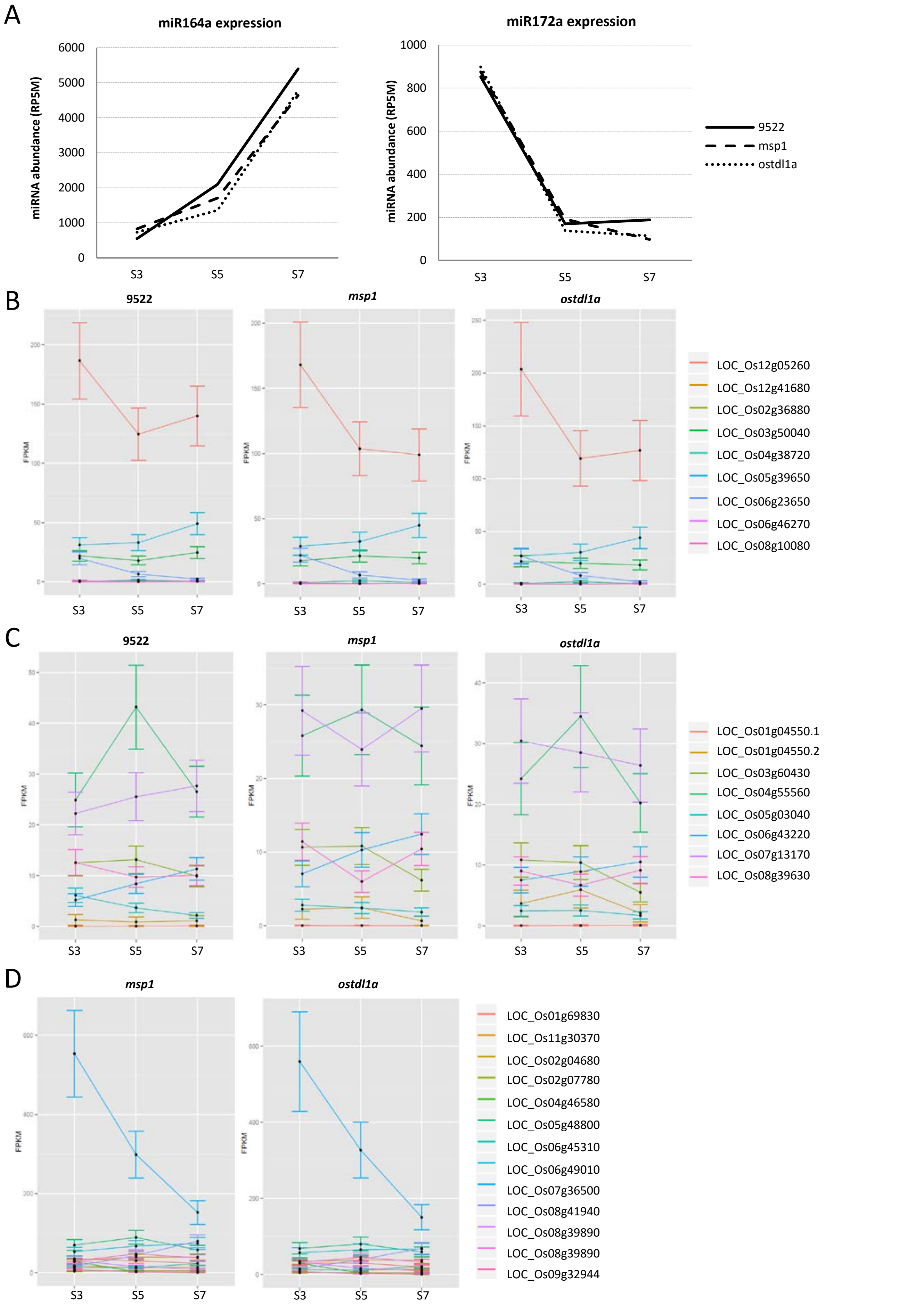

Figure S2. miRNA and target transcript levels in different stages and backgrounds of rice spikelets.
(A) Abundances of miR164 and miR172 across the three stages, representing an average of the three replicates.
(B) Levels of the mRNA targets of miR164 (validated in prior publications – see main text), as measured by RNA-seq. Values represent the average of three replicates. miR164 target gene expression.
(C) Levels of predicted mRNA targets of miR172, measured as in panel B.
(D) Levels of predicted mRNA targets of miR156, measured as in panel B; the wildtype transcript levels are shown in Figure 2C and were not duplicated here.

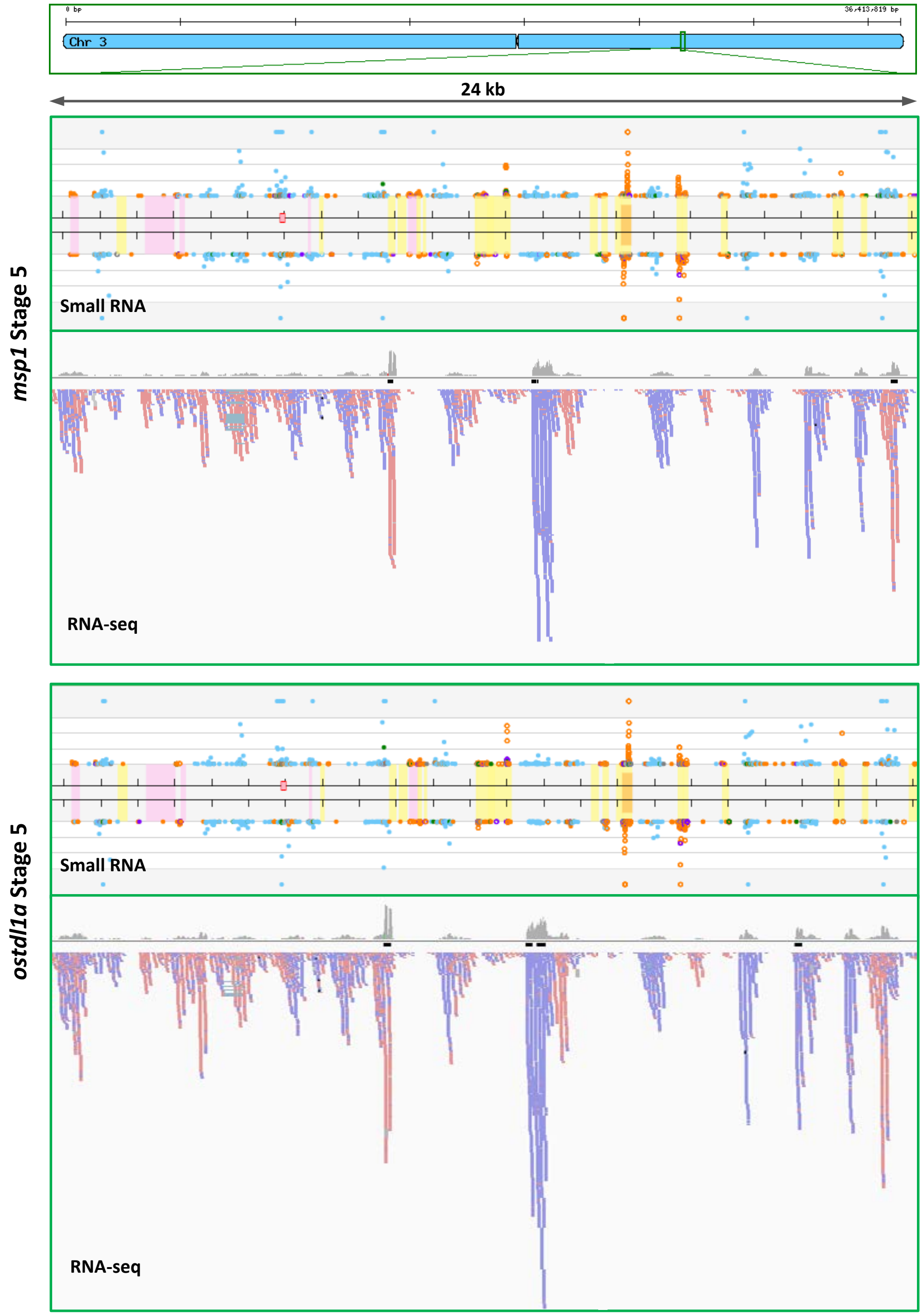

Figure S3. 21-nt phasiRNAs and precursor transcripts were unaffected in both *msp1* and *ostdl1a* mutants at stage 5. A cluster of phasiRNA loci from rice chromosome 3 (approximate location indicated in the image at the very top) were measured in both the *msp1* and *ostdl1a* mutants; the wildtype data are shown in Figure 4 and were not duplicated here, as there is essentially no difference between the mutant and wildtype levels. For the two mutants, the upper image indicates the small RNAs mapped to this 24 kb genomic region. Each dot is a small RNA; light blue are 21-nt sRNAs, green are 22-nt, orange are 24-nt. Yellow shaded regions are predicted DNA transposons; pink shaded regions are predicted retrotransposons; orange shaded regions are inverted repeats. The small pink box is an annotated miRNA. The phasiRNA loci are essentially the distinct blocks of 21-nt sRNAs (light blue). The RNA-seq data is an IGV screenshot; blue bars are top-strand reads, and red bars are bottom strand reads.

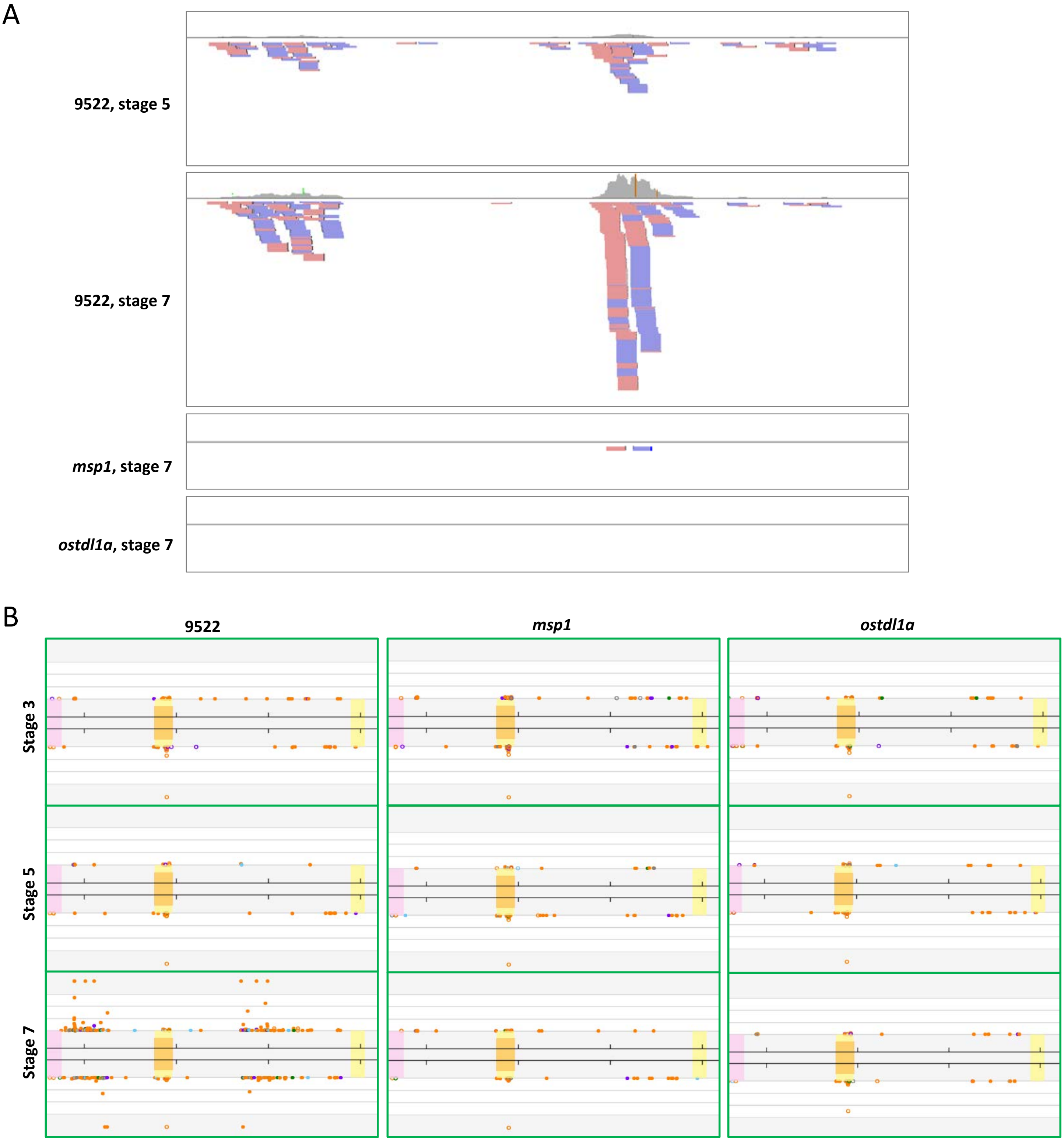

Figure S4. 24-nt phasiRNAs are strongly impacted in stage 7 spikelets of the two rice mutants.
(A) RNA-seq data shown in an IGV-generated screenshot of the locus shown in panel B (Chr6: 25,434,100–25,437,750), for stage 5 and stage 7 spikelets of wildtype and stage 7 of the two mutants. Transcripts producing 24-nt phasiRNAs were highly abundant in Stage 7 of wild type 9522 anthers, but absent in the two mutants.
(B) 24-nt phasiRNA levels increase substantially in stage 7 of wild type rice relative to stage 5, and are almost absent in stage 7 of the two mutants. Each row shows a different stage of spikelets as indicated at the left; the dots and colors are as described in Figure S3. Each column of images shows a different genotype, as indicated at the top.

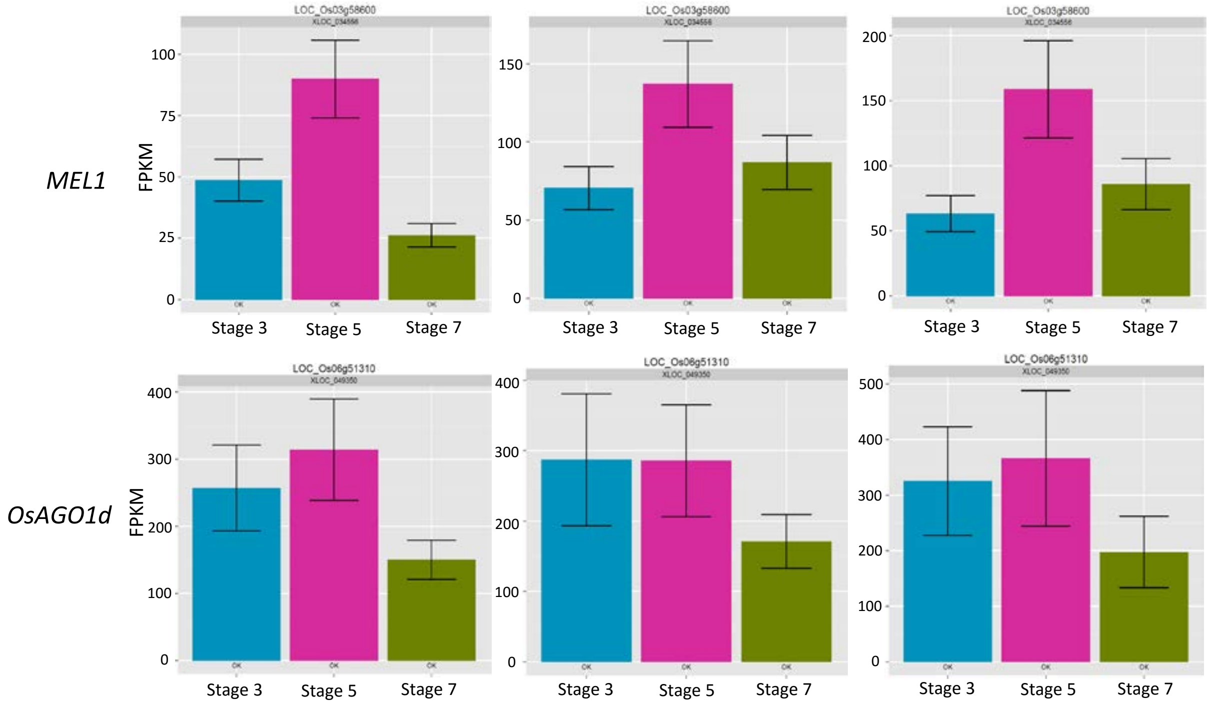

Figure S5. mRNA levels of *MEL1* and *OsAGO1d* in different stages and backgrounds of rice spikelets.

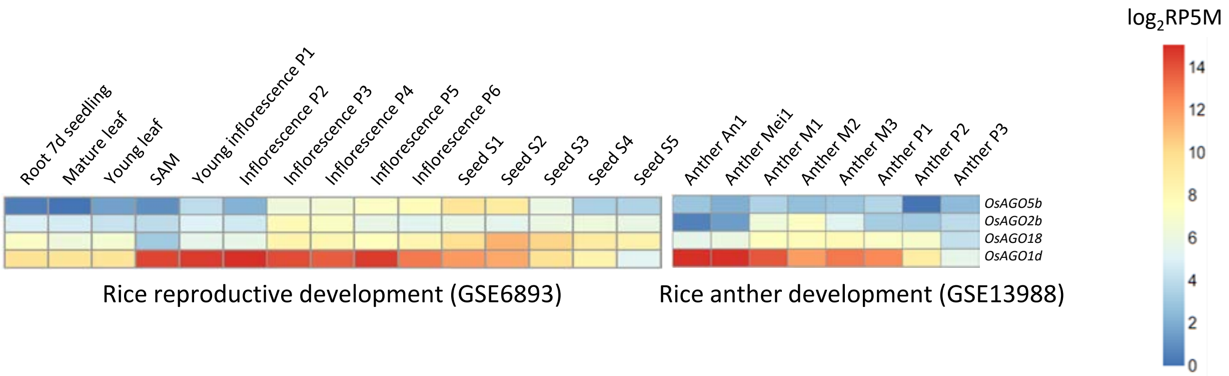

Figure S6. Expression of AGOs in different tissues and developmental stages of rice anthers in public microarray datasets.

Anther-specific expression patterns can be observed for *OsAGO1d*, *OsAGO2b*, *OsAGO18*, but not *OsAGO5b*.
